# Comparison of seventh and eighth edition of AJCC staging system in melanomas at locoregional stage

**DOI:** 10.1186/s12957-019-1669-6

**Published:** 2019-07-25

**Authors:** Pawel Teterycz, Iwona Ługowska, Hanna Koseła-Paterczyk, Piotr Rutkowski

**Affiliations:** 1 Department of Soft Tissue/Bone Sarcoma and Melanoma, Maria Sklodowska-Curie Institute - Oncology Center, Roentgena 5, 02-781 Warsaw, Poland; 2 Early Phase Clinical Trails Unit, Maria Sklodowska-Curie Institute - Oncology Center, Warsaw, Poland

**Keywords:** Melanoma, Staging, AJCC, Sentinel lymph node

## Abstract

**Background:**

The eighth edition of the American Joint Committee on Cancer (AJCC) staging system has been effective since January 2018. It has introduced some major changes in the localized/locoregional melanoma classification. However, it has not been demonstrated how this classification was validated on external, clinical data.

**Patients and methods:**

In this retrospective study, we have included 2474 patients diagnosed with cutaneous melanoma in localized or locoregional stage. They were treated surgically in our Center between years 1998 and 2014. Melanoma-specific and overall survival were calculated for each stage according to TNM7 and TNM8 using Kaplan-Meier estimator.

**Results:**

The melanoma-specific survival rates in our patients were similar to those reported from original cohort used to build TNM8 classification except for stage IIIC (5-year melanoma-specific survival 44.6% vs 51.8%, respectively for TNM7 vs TNM8).

**Conclusion:**

Our study validated the eighth edition of TNM melanoma staging system as a viable tool in prognosis of the long-term survival of patients with localized or locoregionally advanced melanoma on an independent cohort. The new TNM 8 system has brought important improvements in prognostic assessment for melanoma patients. Deeper understanding of the significance of satellite/in-transit lesions may be required.

**Electronic supplementary material:**

The online version of this article (10.1186/s12957-019-1669-6) contains supplementary material, which is available to authorized users.

## Background

Melanoma is an aggressive skin cancer with historically poor prognosis at advanced stage and historically median survival of 7–9 months in the metastatic setting [1]. The prognosis of advanced cases has substantially improved with the introduction of molecularly targeted therapies and immune checkpoint blockage agents. At present, the median survival can be longer than 4–5 years in some selected patient cohorts [2, 3]. The surgery still is the mainstay of therapy at the locoregional stage of the disease. The most recent findings show that incorporation of targeted agents and immunotherapy into the perioperative treatment of less advanced melanoma (i.e., locoregional) may further improve the outcomes of the patients [4, 5]. Therefore, we can expect that the dynamic evolution of metastatic melanoma treatment which took place in the last decade may be replicated in the adjuvant setting.

The eighth edition of the American Joint Committee on Cancer (AJCC) staging system (TNM Classification of Malignant Tumors) has been published at the beginning of 2017 and is effective since January 2018 [6]. It has introduced some major changes in the localized/locoregional melanoma classification. So far, most attention has been paid so far to the changes in stage III of TNM8 as they will have a direct impact on the clinical practice considering the abovementioned rapidly changing landscape of adjuvant treatment options. As a change, the eighth edition of TNM classification is based on an in-depth analysis of the International Melanoma Database and Discovery Platform (IMDDP). IMDDP is a contemporary melanoma database which at the time of building of new TNM included more than 46,000 melanoma cases from 10 institutions in the USA, Europe, and Australia. In comparison with TNM7 database, IMDDP included only cases of cutaneous melanoma diagnosed since 1998, which reflects the modern approach to newly diagnosed melanoma workup, including sentinel lymph node biopsy [6].

There is no doubt that IMDDP is an important source for exploring melanoma patients’ survival and variables which it is affected by. However, it has not yet been shown how new staging system would be validated on external, clinically based data.

In this paper, we present a comparison between the seventh and eighth edition of the AJCC staging based on a large cohort of patients treated for stage I–III melanoma in one reference cancer center.

## Patients and methods

In this retrospective study, we have included 2474 among 2564 consecutive patients diagnosed with cutaneous melanoma in localized or locoregionally advanced stage, who were treated surgically in our Center between years 1998 and 2014. We have excluded from this analysis 90 patients (3.5%) that we were unable to classify in both TNM editions due to missing data. Patients have been undergoing wide local excision, sentinel node biopsy as routine practice in our Center since 1995 as described previously and, furthermore, lymph node dissection in case of positive sentinel node or clinically detected lymph node metastases [7]. The data on demographic features (such as sex and age at diagnosis) and clinicopathological features regarding the primary tumor (Breslow thickness, ulceration, number of mitoses per square millimeter, histological subtype, presence of in-transit lesions, and microsatellites) as well as the nodal disease (clinical detection of metastases, status of sentinel lymph node biopsy (SLNB), tumor location in SLN, and the number of metastatic lymph nodes and diameter of largest of them) were collected in all patients. The survival data in patients which were lost to follow-up after staging in our Center were retrieved from Polish National Cancer Registry.

The overall survival (OS) was calculated from the date of the resection of the primary tumor to the death from any cause. Melanoma-specific survival (MSS) was calculated from the resection of the primary tumor to the death due to melanoma, while patients who died from other causes were censored at the time of death. Patients alive at the date of the last follow-up were censored in both cases.

Discrete characteristics were summarized as numbers and percentages, continuous variables with mean and range in case of normal distribution or with median and interquartile range when distribution was skewed. Kaplan-Meier estimator was used to plot survival curves. Median follow-up time was estimated by the reverse Kaplan-Meier method.

All analyses were performed in the R language environment version 3.5.1 (The R Foundation for Statistical Computing). The data wrangling and visualization was performed with tools from tidyverse and survminer packages [8–10]

## Results

The basic clinicopathological features of our patients are summarized in Table 1. The median follow-up in the whole group reached 12.2 years (95% confidence interval, CI 11.9–12.4). In this period, 976 (39.5%) patients died, 575 (54.8%) of whom due to melanoma. The Kaplan-Meier curves for overall survival according to the stage are shown in Additional file 1: Figure S1.

**Table 1:** Patients characteristics

Variable		*N* (percentage)
Patient sex	Female	1382 (54.82%)
	Male	1092 (43.32%)
Ulceration	Absent	1206 (47.84%)
	Present	1017 (40.34%)
	Unknown	251 (9.96%)
Microsatellites	Absent	2272 (90.12%)
	Present	98 (3.89%)
	Unknown	104 (4.13%)
In-transit	Absent	2366 (93.85%)
	Present	85 (3.37%)
	Unknown	23 (0.91%)
T feature	1a	135 (5.36%)
	1b	318 (12.61%)
	1, unable to stage otherwise	52 (2.06%)
	2a	416 (16.5%)
	2b	165 (6.55%)
	2, unable to stage otherwise	43 (1.71%)
	3a	278 (11.03%)
	3b	359 (14.24%)
	3, unable to stage otherwise	47 (1.86%)
	4a	145 (5.75%)
	4b	421 (16.7%)
	4, unable to stage otherwise	39 (1.55%)
	Unknown	56 (4.09%)
N feature	Negative	1560 (61.88%)
	1a	252 (10%)
	1b	103 (4.09%)
	1c	45 (1.79%)
	2a	116 (4.6%)
	2b	93 (3.69%)
	2c	32 (1.27%)
	3a	38 (1.51%)
	3b	129 (5.12%)
	3c	70 (2.78%)
	Unknown	36 (3.29%)
Sentinel lymph node biopsy status	Negative	1711 (67.87%)
	Not conducted	292 (11.58%)
	Positive	471 (18.68%)
Largest metastatic deposit in sentinel lymph node	Not applicable	1599 (64.63%)
	Clinical (palpable)	292 (12.9%)
	Unknown	212 (9.37%)
	< 1 mm	112 (4.53%)
	≥ 1 mm	259 (10.42%)
Year of diagnosis	Median	2004
	Interquartile range	2002–2008
Age	Mean	51.69
	Range	14–94
Breslow thickness (mm)	Median	2.5
	Interquartile range	1.2–4.1

**Fig. 1:**
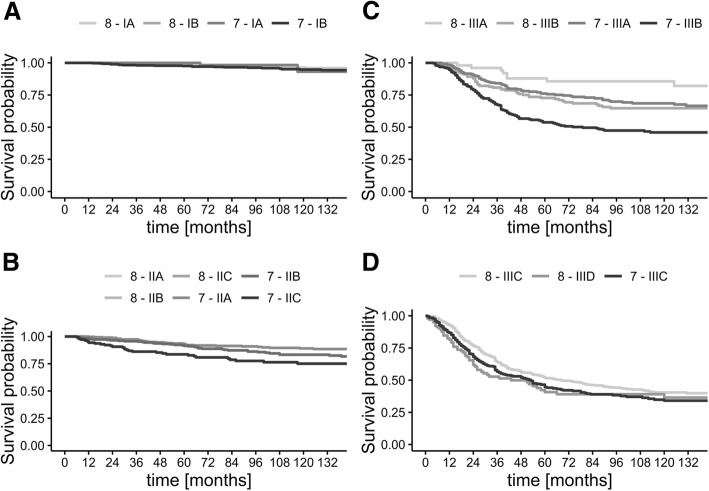
Kaplan-Meier curves of melanoma-specific survival according to both TNM version 7 and TNM version 8. **a** Stage I. **b** Stage II (note that the curves for TNM7 and TNM8 are overlapping). **c** Substages IIIA and IIIB. **d** Substages IIIC and IIID

The stage for the patients according to both TNM7 and TNM8 is presented in Table 2 while the melanoma-specific and overall survival curves for each pathological stage are presented in Fig. 1 and Additional file 1: Figure S2 respectively. When considering the stage I group, it is worth to note that none of the patients has been upstaged from IA to IB, yet 107 (23.1%) of the patients who were deemed to be stage IB in previous TNM system have been downstaged to IA. This change did not influence the survival rates in both stages IA and IB in a significant way, but it allowed for less traumatic surgical treatment. It is also important to mention that exclusion of the mitotic rate from the staging system simplified the process and allowed to categorize patients even with poor histopathological report from the primary resection—in the stage I of TNM7, 239 (30.7%) cases could not be classified into A/B substages, while in the TNM8, there were only 27 (3.5%) such cases. While this should not impact patients treated in reference centers, it may be an important consideration in case of patients after primary treatment in less specialized facilities. As expected, there are no differences in classification of patients in the stage II group.

**Table 2:** Raw numbers of patients staged according to both TNM version 7 and TNM version 8. In case of differences between classifications, the font is italics

		TNM version 8											
		I	IA	IB	II	IIA	IIB	IIC	III	IIIA	IIIB	IIIC	IIID
TNM 7	I	27	*212*										
	IA		76										
	IB	*4*	*107*	351									
	II				73								
	IIA					308							
	IIB						258						
	IIC							144					
	III								74			*5*	
	IIIA								*7*	51	*82*	*112*	
	IIIB								*7*		79	*168*	
	IIIC											229	*100*

The most changes have been observed in the stage III group. While the direct translation of TNM7 into TNM8 is not possible, it would be helpful to compare prognosis between those two classifications. It is important to mention that the new stage III is a very diverse group of patients. The subgroup IIIA has excellent prognosis—according to our data even better than some patients with thick melanoma without lymph node involvement (i.e., substage IIC). To the contrary, subgroup IIID has a dramatically poor prognosis with only 26% 5-year survival rate.

Exact 5- and 10-year survival rates, both for melanoma-specific and overall survival, are presented in Table 3 and Additional file 1: Table S1 respectively. It is worth noting that in our dataset, stages IIIC and IIID are not significantly different.

**Table 3:** Five- and 10-year melanoma-specific survival rates according to TNM version 7 and TNM version 8 stage

	TNM version 8				TNM version 7			
Stage	5 year	95% CI	10 year	95% CI	5 year	95% CI	10 year	95% CI
I	98.20	97.20–99.10	95.20	93.50–96.9	98.20	97.20–99.10	95.20	93.50–96.9
IA	98.46	97.24–99.69	96.37	94.34–98.44	100	100–100	93.13	83.33–100
IB	97.98	96.51–99.47	94.46	91.79–97.2	97.57	96.16–99	94.77	92.5–97.09
II	90.50	88.50–92.70	84.70	82.00–87.50	90.50	88.50–92.70	84.70	82.00–87.50
IIA	92.86	89.96–95.85	89.07	85.41–92.89	92.86	89.96–95.85	89.07	85.41–92.89
IIB	91.47	88.04–95.03	83.31	78.46–88.46	91.47	88.04–95.03	83.31	78.46–88.46
IIC	83.63	77.58–90.16	75.11	67.53–83.54	83.63	77.58–90.16	75.11	67.53–83.54
III	59.20	55.90–62.70	51.00	47.50–54.90	59.20	55.90–62.70	51.00	47.50–54.90
IIIA	87.86	79.21–97.46	85.61	76.28–96.09	76.13	70.89–81.76	68.53	62.51–75.11
IIIB	72.7	65.86–80.25	64.75	57.16–73.34	53.76	47.62–60.68	45.93	39.64–53.22
IIIC	51.77	47.31–56.65	40.48	35.77–45.82	44.59	38.93–51.07	34.07	28.27–41.06
IIID	40.72	31.09–53.34	36.49	26.7–49.86	NA		NA	

*CI* confidence interval

**Fig. 2:**
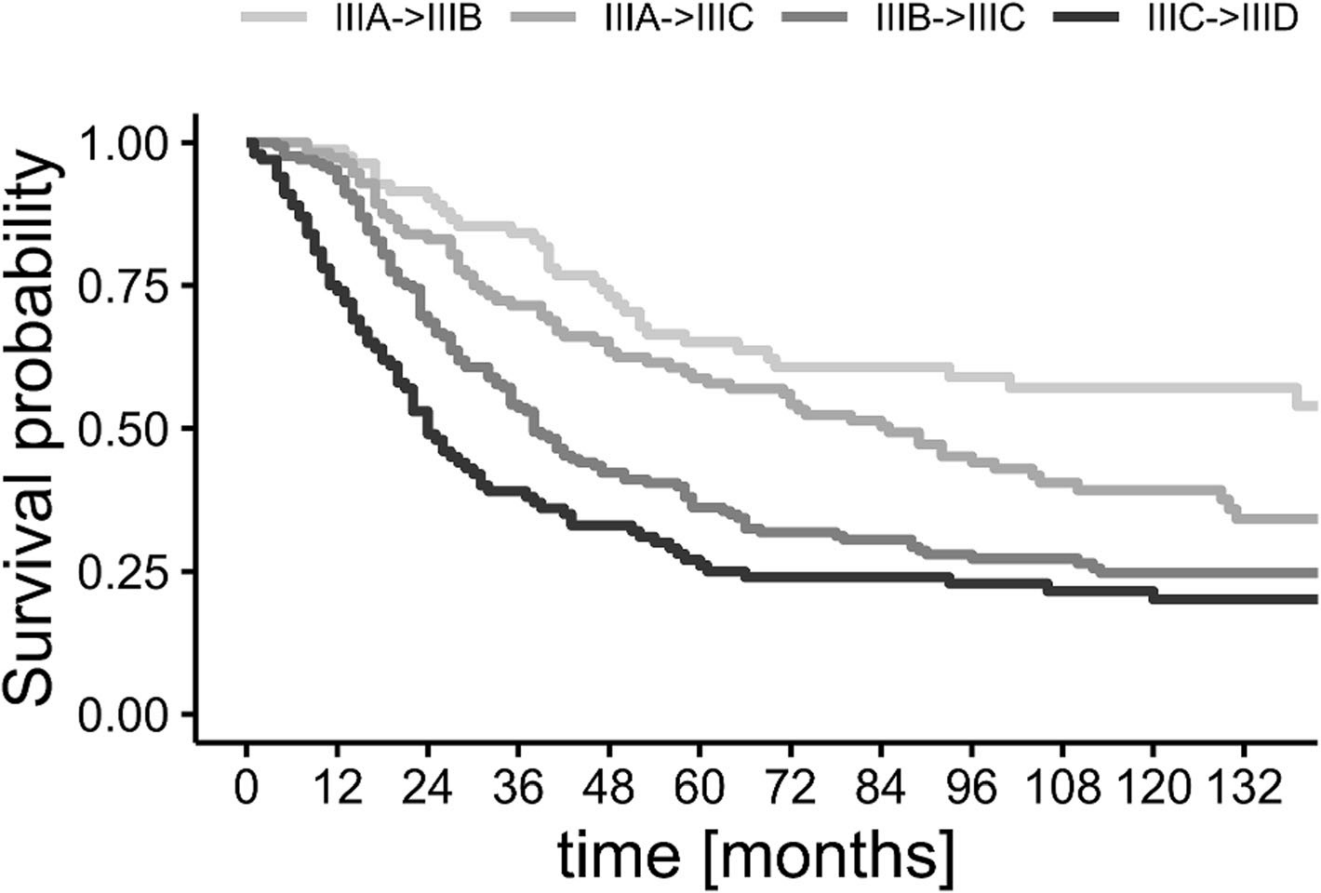
Kaplan-Meier curves for stage III patients whose TNM7 and TNM8 substage differed. The curves are described in fashion of [substage in TNM7]- > [substage in TNM8]

Figure 2 shows the survival rates in stage III patients who would be classified in different substage in TNM8. Indeed, those patients who were upstaged in the new classification were also at much higher risk of spread of the disease and death due to melanoma than it was anticipated in TNM7.

## Discussion

In this study, we have shown that the TNM8 system is validated well by independent data external in regard to IMDDP. We have demonstrated that MSS rates are generally similar to the original modeling cohort.

To our knowledge, this is the largest validation of TNM8 system on the homogenous data at stages I–III not included in the IMDDP. All of the patients in this cohort were treated in a uniform fashion in a single, reference center, adhering to the guidelines applicable at the time of melanoma treatment. As this study is based on the experience of one center, the source medical documentation was available for all patients. This might not be possible in case of register-based studies. The information obtained from the National Cancer Registry allowed for the accumulation of accurate data on the survival of our patients with an extended observational period (even as long as 19 years). The inclusion criteria in this analysis are similar to the ones used in building of the TNM8; therefore, it is reasonable to assume comparability of our results with the original cohort. In contrast, the patients’ cohort in this analysis is mainly uniform ethnically and the underlying gene polymorphisms and sun exposure patterns may affect the external validity of our results.

When considering stage I patients, the MSSR reported by Gershenwald and Scolyer are always in the 95% CI of our analysis [11]. In stage II, only the subgroup IIB showed slightly better 5-year MSSR in our group. The only real differences in survival rate comparison are visible in stages IIIC and IIID—our stage IIIC patients did not show as favorable prognosis as the ones reported by Gershenwald (10-year MSSR 40.5%, CI vs. 60% 10-year MSSR), whereas in the stage IIID, prognosis was (numerically) 12 percentage points better than in the IMDDB cohort (36.5% 10-year MSSR, CI vs. 24% 10-year MSSR). Indeed, our data do not fully support the differentiation between IIIC and IIID. Nevertheless, we did observe similar trends in MSS as the Gershenwald et al. In Cox’s proportional hazard model, hazard ratio for the comparison of stage IIID vs. IIIC reached 1.32 (95% confidence interval 0.98–1.78, *p* = 0.06).

The comparison between TNM7 and TNM8 shows better categorization while using the newer one. Especially, the strict selection of thin melanoma with only up to three clinically occult metastatic lymph nodes (IIIA) allowed to indicate a N+ group with good predicted survival (5-year MSSR of 87.9%) which was not highlighted in the TNM7 (76.1% 5-year MSSR). At the same time, it is worth to mention that the number of patients with IIIA disease dropped drastically in our cohort from 252 to 51 (drop of 80%). The small improvement in the prognostic value in subsetting of T1 category (not statistically significant) can be also found in the new stage I.

In our analysis 704 (28.5%) of patients were restaged—41.6% of stage I and 50.1% of stage III. As has been pointed out, this can have an impact on the analysis of some of the available results of adjuvant clinical studies [12]. At the same time, lack of clinical benefit from completion lymphadenectomy after positive SLNB and probable gradual refrain from this procedure in the near future may also limit the usefulness of the current TNM in the adjuvant setting [13]. Incorporation of sentinel node tumor burden might help to alleviate this problem in the future [14]. On the other hand, TNM8 can be an important tool for individualized approach for adjuvant therapy when complete workup is performed. Eggermont et al. recently showed that current classification has a significant prognostic (however not predictive) value regarding relapse-free survival when anti-PD1 therapy is applied in adjuvant setting. Furthermore, the abovementioned study confirmed excellent prognosis in the current stage IIIA disease [15].

The efforts to validate TNM8 system have been taken recently by some authors [16–19]. Verver et al. focused on the stage I melanoma in a registry-based study and concluded that TNM8 can help in better selection of high-risk T1 melanomas which is concordant with our observations [16]. In a population-based study by Crocetti et al., they showed average agreement between stage I subgroups, very good for stage II and very poor for stage III [17]. This study also concentrated mainly on the early-stage melanoma. Isaksson et al. focused on stage III cases and reported similar MSSR for subgroups IIIA, IIIB, and IIIC as we do. Contrary to our findings, patients in the group IIID fared worse than reported in the original IMDDP cohort [19].

Our study has limitations. First of all, the relatively small number of patients in group IIID can influence the reproducibility of the results. Moreover, the incomplete understanding of the biology of satellitosis/in-transit metastases warrants further research in this regard. Secondly, the lack of full data on the size of the largest metastatic deposit in sentinel lymph node makes it difficult to assess the importance of this factor in our cohort.

In conclusion, our study generally validated the new, 8th edition of TNM melanoma staging system in the regard of long-term survival of patients with localized or locoregionally advanced melanoma. The cases of high-risk patients, such as stage IIIC or IIID, may need careful reexamination in larger, preferably multi-center, cohort. It has also compared TNM8 with the previous, 7th edition of this system. We have showed similar survival rates to those reported in the IMDDP database. The new TNM8 system has brought important improvements in the prognostic assessment for melanoma patients and may help in clinical decision-making. However, it may not be completely well suited to meet the requirements of rapidly changing state of the art of melanoma treatment.

## Additional file

Additional file 1: Figure S1. Overall survival according to: A. TNM version 7 B. TNM version 8. Figure 2. Kaplan-Meier curves of overall survival according to both TNM version 7 and TNM version 8. A. Stage I. B. Stage II (note that curves for TNM7 and TNM8 are overlapping) C. Substages IIIA and IIIB. D. Substages IIIC and IIID. Table S1. Five- and 10-year overall survival rates according to TNM version 7 and TNM version 8 stage. (PDF 120 kb)

## Data Availability

Not applicable.
