# Supplementary material for: Comparison of seventh and eighth edition of AJCC staging system in melanomas at locoregional stage
Source: World J Surg Oncol. 2019 Jul 25;17:129. doi: 10.1186/s12957-019-1669-6 (PMC6657085; doi:10.1186/s12957-019-1669-6)
Supplement: Supplementary file 1 — Figure S1. Overall survival according to: A. TNM version 7 B. TNM version 8. Figure 2. Kaplan-Meier curves of overall survival according to both TNM version 7 and TNM version 8. A. Stage I. B. Stage II (note that curves for TNM7 and TNM8 are overlapping) C. Substages IIIA and IIIB. D. Substages IIIC and IIID. Table S1. Five- and 10-year overall survival rates according to TNM version 7 and TNM version 8 stage. (PDF 120 kb) [file 12957_2019_1669_MOESM1_ESM.pdf]

Comparison of 8th and 7th edition of AJCC staging system in melanomas at locoregional stage - supplementary materials

Teterycz P, Ługowska I, Koseła-Paterczyk H, Rutkowski P

Figure 1

Overall survival according to: A. TNM version 7 B. TNM version 8.

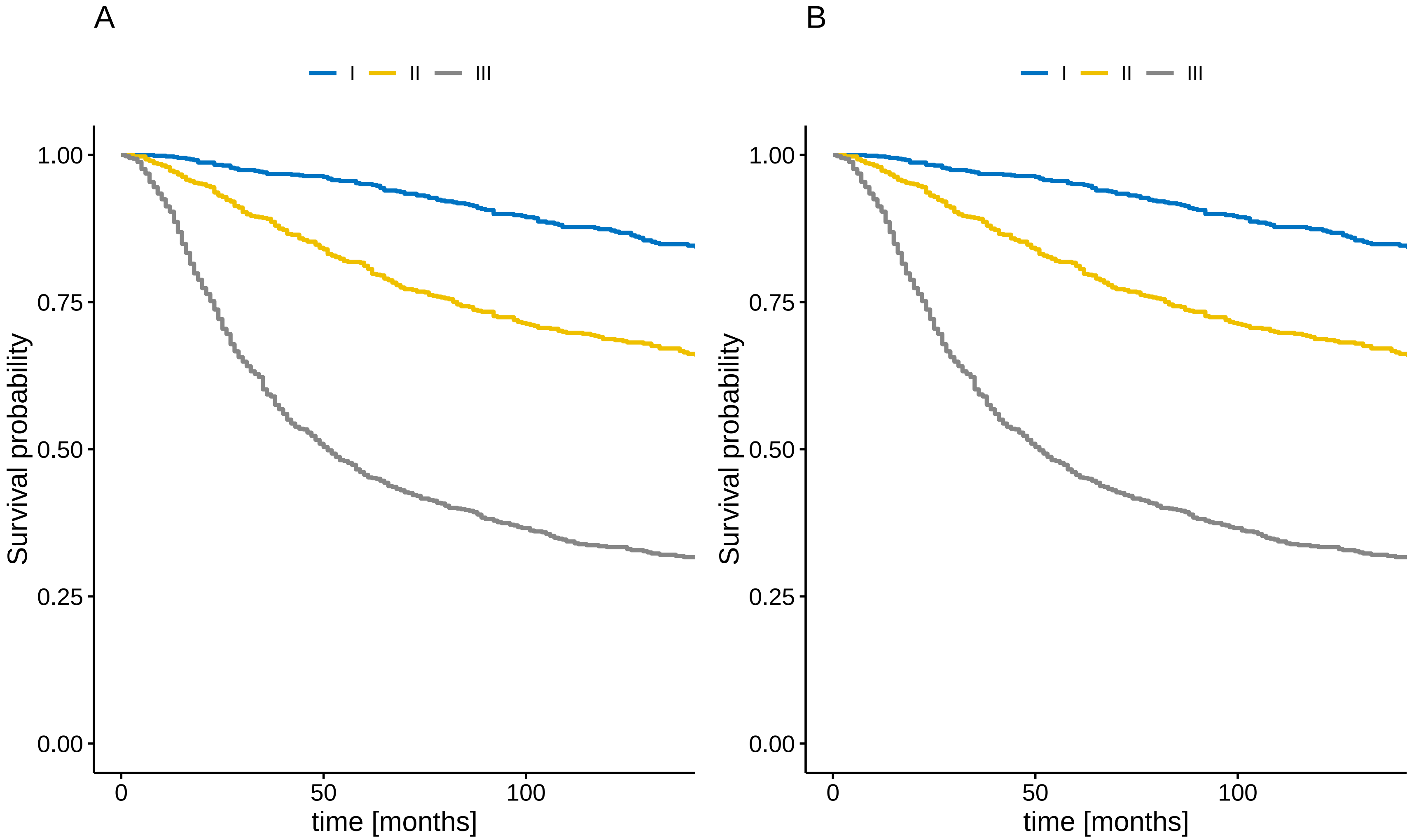

Figure 2

Kaplan-Meier curves of overall survival according to both TNM version 7 and TNM version 8. A. Stage I. B. Stage II (note that curves for TNM7 and TNM8 are overlapping) C. Substages IIIA and IIIB. D. Substages IIIC and IIID.

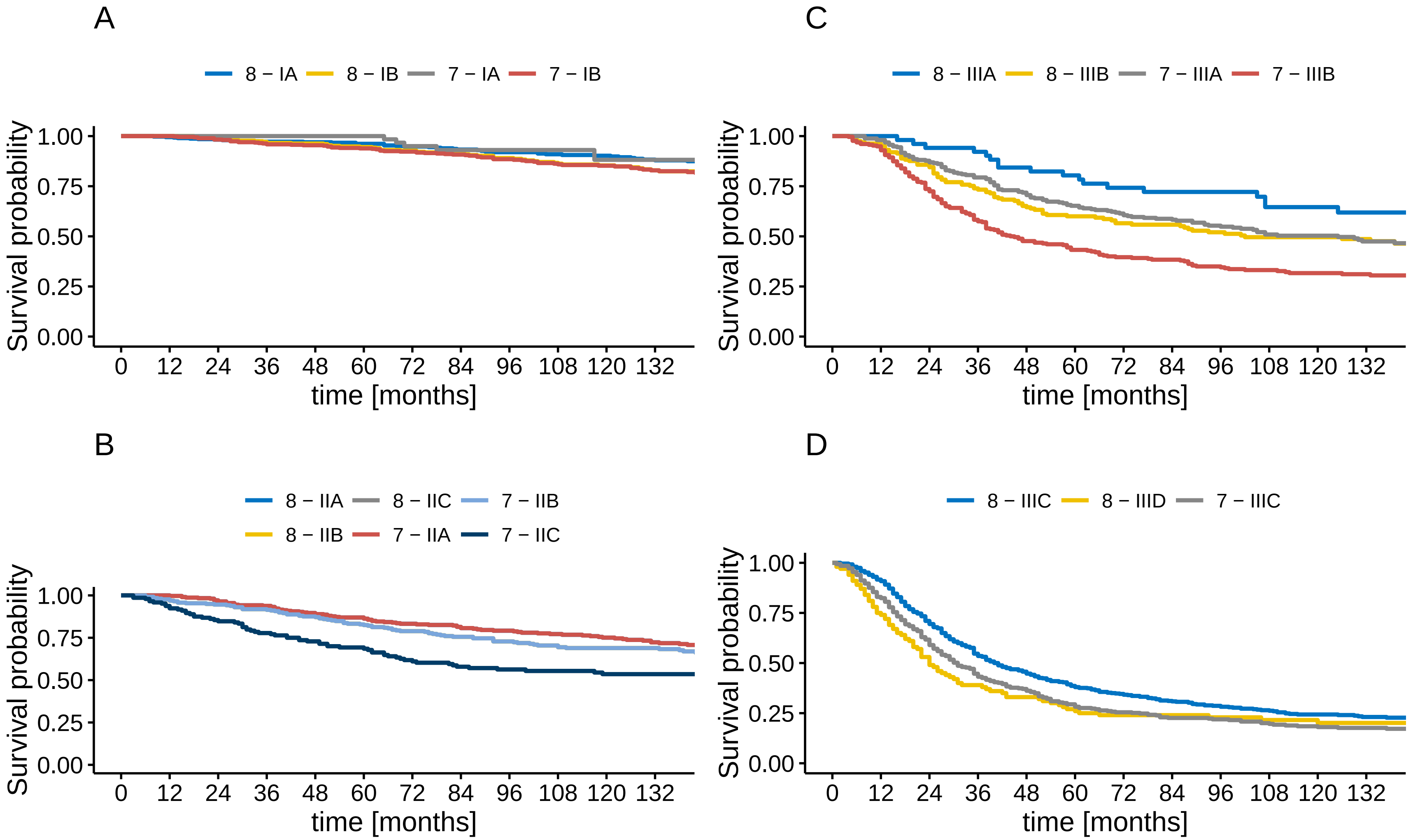

Table 1

5- and 10-year overall survival rates according to TNM version 7 and TNM version 8 stage.

Stage	TNM8 5 year	TNM8 5 year 95%CI	TNM8 10 year	TNM8 10 year 95%CI
I	95.00	93.50 - 96.60	87.40	84.90 - 89.90
IA	96.15	94.26 - 98.08	90.21	87.1 - 93.43
IB	94.54	92.18 - 96.96	85.39	81.47 - 89.5
II	81.20	78.40 - 84.00	68.70	65.4 - 72.20
IIA	86.28	82.51 - 90.23	75.08	70.2 - 80.29
IIB	82.48	77.96 - 87.27	68.92	63.29 - 75.05
IIC	68.53	61.32 - 76.6	53.52	45.69 - 62.69
III	45.70	42.50 - 49.00	33.40	30.30 - 36.70
IIIA	80.39	70.2 - 92.06	64.58	52.18 - 79.92
IIIB	59.98	52.83 - 68.09	49.57	42.16 - 58.28
IIIC	37.99	34.01 - 42.44	24.33	20.76 - 28.53
IIID	26	18.68 - 36.19	20.12	13.45 - 30.11

Stage	TNM7 5 year	TNM7 5 year 95%CI	TNM7 10 year	TNM7 10 year 95%CI
I	95.00	93.50 - 96.60	87.40	84.90 - 89.90
IA	100	100 - 100	88.16	77.6 - 100
IB	93.86	91.68 - 96.09	85.22	81.73 - 88.85
II	81.20	78.40 - 84.00	68.70	65.4 - 72.20
IIA	86.28	82.51 - 90.23	75.08	70.2 - 80.29
IIB	82.48	77.96 - 87.27	68.92	63.29 - 75.05
IIC	68.53	61.32 - 76.6	53.52	45.69 - 62.69
III	45.70	42.50 - 49.00	33.40	30.30 - 36.70
IIIA	65.23	59.58 - 71.41	50.32	44.19 - 57.3
IIIB	43.23	37.54 - 49.78	31.64	26.31 - 38.07
IIIC	28.19	23.72 - 33.51	18.06	14.24 - 22.91
IIID	NA	NA	NA	NA
